# Examining the relationship between self-efficacy, career development, and subjective wellbeing in physical education students

**DOI:** 10.1038/s41598-024-59238-6

**Published:** 2024-04-12

**Authors:** Yikeranmu Yiming, Bing Shi, Sumaira Kayani, Michele Biasutti

**Affiliations:** 1 Physical Education School, Shaanxi Normal University, 710000 Xi’an, China; 2 Department of Applied Psychology, University of Toronto, Toronto, Canada; 3 Department of Philosophy, Sociology, Education and Applied Psychology (FISPPA), University of Padova, 35139 Padova, Italy

**Keywords:** Psychology, Human behaviour

## Abstract

We investigated the relationship between self-efficacy and career development via subjective well-being of students majoring in physical education. Life satisfaction, positive affect, and negative affect were the componennts of subjective well-being. Participants were the 1381 adolescents with major in physical education with an age range of 18–22 years (Mage = 19.5 ± 1; females = 34.76%). Hayes PROCESS model was used to develop a multiple mediation model. The results suggest that higher self-efficacy leads to better career development. Further, a significant mediating role was played by negative and positive affect in case of the relationship between self-efficacy and career exploration, but life-satisfaction is not significant mediator. Conversely, life satisfaction and positive affect are significant mediators between self-efficacy and career adaptability but negative affect is not. The findings suggest that self-efficacy and subjective well-being benefit career development of adolescents in the physical education field.

## Introduction

Career development means an individual’s selection, entry, and growth in the professional, educational, and other essential facets of life, with the ultimate objective of achieving career-related goals^1^. According to career construction theory, career development can be explained by several factors such as career knowledge, career identity, career commitment, career decision making, career exploration, and career adaptability^2^. The current study focuses on career exploration and career adaptability because these are considered important to capture different aspects of an individual’s career journey and provide insights into their ability to navigate and adapt to changing work environments^3,4^. Career exploration is choosing a career according to one’s beliefs, goals, and interests^5–7^. Career adaptability is a psychosocial concept that focuses on a person’s ability to manage developmental tasks to prepare for future professional careers^8^.

The career exploration and adaptability is a crucial issue among school adolescents as they undergo a critical phase during their career exploration and adaptability^2^. The school adolescents in the field of physical education feel not very well prepared for choosing and adapting their career because of an inclination towards reevaluating reality, realizing their thoughts, and developing early professional goals^9^. It is perceived that adolescents majoring in physical education or sports usually possess more career anxiety compared to others^10^ because they do not usually possess a positive attitude towards career, decision, exploration and adaptation or think about choosing the alternative professions^11^. The anxiety and pressure hinders their future job planning and decision-making and diminishes their desire to engage in physical activities and sports training^9^. Hence, it is necessary to investigate the factors affecting the career exploration and adaptability of physical education students to provide proper guidance and establish competitive, widespread, and extensive physical education systems for adolescents (Yiming et al. 2023).

Social cognitive theory (SCT), developed by Albert Bandura, emphasizes the importance of self-efficacy beliefs in shaping an individual’s behavior and career choices (Bandura, 1997; Biasutti and Concina 2018; Biasutti et al. 2021). The Social-Cognitive Career Theory (SCCT)—Satisfaction Model built on Bandura’s social cognitive theory is a theoretical framework that explains how self-efficacy beliefs, outcome expectations, personal goals, contextual influences, and subjective well-being influence and shape an individual’s career development and job satisfaction^12–14^. Hence, theory could provide a strong theoretical evidence on how self-efficacy is related to an individual’s career development, and subjective wellbeing. For example, SCCT suggests that self-efficacy beliefs influence career development by shaping individuals’ career-related behaviors and decisions as individuals with high self-efficacy are more likely to set ambitious career goals and persist in the face of challenges (Lent et al., 1994). A study by Lent et al. (2000) found that self-efficacy was positively associated with career exploration and career decision-making self-efficacy among college students, highlighting the role of self-efficacy in career development.

Self-efficacy, as a cognitive variable positively affects career-related behaviors of adolescents in both the general and physical education fields^10,12,15,16^. Stead, LaVeck^17^ examined 18 research studies on 6339 adolescents and highlighted a significant association between career adaptability and self-efficacy. Another recent meta-analysis has found that students’ self-efficacy significantly increased career exploration among 34,969 college students^18^. Chan^16^ investigated the association between self-efficacy and career-related variables such as career self-efficacy, career beliefs, and social support and found in his series of research that self-efficacy positively predicted career choice and beliefs of college sports students in Taiwan^10,15,16^. Another longitudinal study on 24,273 high school adolescents in China explored the positive impact of career decision-making self-efficacy on their career exploration^19^.

Previous research has considered career-related self-efficacy and neglected the role of generalized self-efficacy in promoting an individual’s career development, except for one recent study in South Asia^20^. Hence, it is necessary to establish an association between generalized self-efficacy and career development to provide insight to career counsellors, organizational psychologists, and positive psychologists for developing future interventions. Moreover, past research has focused on school and college adolescents for conducting their research^18,19^ and the studies on physical education students are scarce. Therefore, we expect a significant positive effect of generalized self-efficacy on the career exploration and adaptability of these students^20^.

Recent research has considered several mediators between self-efficacy and career development suggesting a mechanism between the two variables. This brought our attention towards possible mediators which may play their role in transferring the effect of self-efficacy to career development. A possible mediator between self-efficacy and career-related behaviors could be subjective well-being which refers to an individual’s cognitive and affective assessments of his life^21–23^. The two most significant components of subjective well-being are cognition and affect. The cognitive component of subjective well-being is related to life satisfaction which acknowledges that well-being is not only determined by objective circumstances but also influenced by individual perceptions, values, and interpretations of one’s life experiences^23,24^. Moreover, the level of subjective well-being is indicated by the levels of positive and negative components^21,22^.

Several studies demonstrated the association between self-efficacy and subjective well-being and evidenced that self-efficacy is crucial in promoting adolescents’ subjective well-being^20^. SCCT proposes that self-efficacy is related to subjective well-being, as individuals who believe in their ability to achieve their goals tend to experience greater satisfaction and happiness in life (Lent and Brown 2013). For example, research by Lent and Brown (2006) found that self-efficacy was positively associated with job satisfaction and life satisfaction among individuals, supporting the idea that self-efficacy contributes to subjective well-being. Also, high self-efficacy was connected with higher subjective well-being^20,25^, and low self-efficacy was associated with lower levels of subjective well-being^26^. In the context of life satisfaction, individuals’ beliefs, self-efficacy, and observational learning can significantly impact overall well-being and satisfaction with life leading to better career choices and actions (Bandura 1997). Further, it is believed that individuals with high self-efficacy would turn up with high levels of life satisfaction^27–29^, possessing a positive attitude and avoiding negative behavior^20,29^.

Moreover, SCCT also suggests a positive association between one’s subjective wellbeing and a career that aligns with one’s values and interests. Individuals who pursue careers that provide opportunities for growth and development are more likely to experience a sense of fulfillment and purpose. Conversely, those who find fulfillment and purpose in their work are often motivated to seek out opportunities for growth and development in their careers. This relationship suggests a mutually reinforcing cycle where career growth and development contribute to a sense of fulfillment and purpose, which in turn motivates further career development (Lent and Brown 2013). For instance, research from the middle east, exhibits that life satisfaction, positive affect and avoiding negative behavior have positive impact on career choice and adaptability of individuals^30^. Similarly, life satisfaction and positive attitude towards life are connected with better career choise and higher career adaptability^30^. A recent study among Italian students has also found a positive link between life satisfaction and career related behaviors^31^.

Considering the above theoretical background on the positive association between self-efficacy, life satisfaction, positive affect, career exploration, and career adaptability in several societies, life satisfaction, positive affect and avoiding negative behavior might explain the relationship between self-efficacy and career development among adolescents, the following hypotheses were generated:

(*H1*) Self-efficacy would positively affect career exploration.

(*H2*) Life satisfaction would significantly mediate between self-efficacy and career exploration.

(*H3*) Positive/negative affect would significantly mediate between self-efficacy and career exploration.

(*H4*) Self-efficacy would positively affect career adaptability.

(*H5*) Life satisfaction would significantly mediate between self-efficacy and career adaptability.

(*H6*) Positive/negative affect would significantly mediate between self-efficacy and career adaptability.

## Method

### Participants

The research participants were adolescents majoring in physical education from Xi’an and Xinjiang Uygur Autonomous Region of China. Students of physical education are considered more anxious about their careers as they do not have another option to choose career from^10,11^. The anxiety or pressure exerted on physical education students may lead to reduce their ability of career decision making, career choice and adaptability^9^ and therefore, we have chosen the adolescents majoring in physical education. Data was collected from physical education students through questionnaires. Convenient sampling technique was used to recruit participants. One thousand five hundred questionnaires were distributed to students who were convenient to approach. Fourteen hundred and four (1404) questionnaires were returned. Twenty-three cases were removed based on missing values and Mahala Nobis distance and finally, 1381 were the number of participants with a valid response with a response rate of 92%. There were 901 (65.24%) males and 480 (34.76%) females with the age range of 18–22 years old.

### Measures for data collection

#### Generalized self-efficacy

According to Bandura^34^, generalized self-efficacy refers to a person’s belief in their ability to effectively cope with a variety of challenging situations and to achieve desired outcomes in different areas of life. It reflects a broad and generalized sense of confidence in one’s capabilities to handle diverse tasks and challenges. Generalized self-efficacy (GSE) was measured by using Chinese version of GSE scale which has already validated in Chinese setting^35^. The authors adapted Chinese version of the GSE from traditional typical Chinese language used in Hong Kong and Taiwan (Zhang and Schwarzer 1995). They translated the scale into simplified Chinese language of mainland China, and validated it in the region. The scale contained 10 items rated on 4-point Likert scale with a range of 1 = not at all true and 4 = exactly true. The example items are “I can solve most problems if I invest the necessary effort”, and “I am confident that I could deal efficiently with unexpected events”. The sum score was calculated having a minimum score of 10 showing least self-efficacy, and a maximum score of 40 indicating highest self-efficacy. The scale had two factors containing 5 items in each. However, unidimensional scale is considered for the current study. Further, the tool is valid in terms of all significant fit indices such as X^2^/df = 4. 31, CFI = 0.93, TLI = 0.91, RMSEA = 0.076, SRMR = 0.043, indicating the construct validity. The internal consistency of the scale is 0.92.

#### Career adaptability

Career adaptability refers to an individual’s ability to effectively navigate and respond to changes and challenges in their career path^4^. It encompasses several key components such as concern, control, curiosity, and confidence^36^. The Career Adaptability Scale with Mandarin translation was used to assess career adaptability, and the scale was already validated in Chinese setting^37^. It consists of four subscales, each measuring one of the four aspects of professional flexibility (control, curiosity, confidence, and concern), with 3 items in each factor. However, we have used the scale as unidimensional for the current study. The example items are, “Realized the study and work path I had to choose”, and “take responsibility for my actions”. All items were rated on 5-point Likert scale of 1 (strongly disagree) to 5 (strongly agree). The total score was calculated with 12 exhibiting less adaptability and 60 showing higher adaptability. Cronbach’s alpha for the scale was 0.96. Further, the tool with 4 factor model is valid in terms of all significant fit indices such as X^2^/df = 3.27, CFI = 0.95, TLI = 0.93, RMSEA = 0.08, SRMR = 0.051, indicating the construct validity.

#### Career exploration scale

Career exploration is the process by which individuals gather information about occupations, industries, and educational pathways to make informed decisions about their career goals and aspirations^38^. The scale for exploring careers was adapted from a prior study in which the tool has already validated in Chinese setting^39^. The scale of career exploration is a subcomponent of the “career and talent development self-efficacy measure”. It is the unidimensional scale containing a six-item for self-measure of career exploration. The ratings range from 1 (extremely lacking in confidence) to 6 (extremely confident). The example items are, “cultivate my interests according to the career I choose”, and “understand the relationship between the present campus life, further study and future career”. According to the scale, a score of 6 indicates a low degree of career exploration, while a score of 30 indicates a high level. The sum of all the elements on the scale represents the final score. The scale has been utilized in its Chinese form, which has already been validated. Cronbach’s alpha for the scale was 0.88. Further, the tool with one factor model is valid in terms of all significant fit indices such as X^2^/df = 4.45, CFI = 0.91, TLI = 0.89, RMSEA = 0.071, SRMR = 0.049, indicating construct validity of the scale.

### Subjective well being

Subjective well-being was measured by using life satisfaction, positive and negative affect.

### Life satisfaction

Life satisfaction is an individual’s overall evaluation of the quality of their life, encompassing various domains such as work, relationships, health, and personal fulfillment^21^. The Leung and Leung^40^’s life satisfaction scale, which has five items, was used to measure life satisfaction. The authors have already validated the tool in Chinese setting by Leung and Leung^40^. Each response is given a rating on a Likert scale of 1–7, with 1 showing less agreement and 7 representing higher agreement. It includes statements such as “In most ways, my life is close to my ideal”, and “I am satisfied with my life”. Excellent psychometric qualities characterize the measure^40^ with a Cronbach alpha value of 0.78. A total score ranged between 5 and 35. Further, the tool with one factor model is valid in terms of all significant fit indices such as X^2^/df = 2.98, CFI = 0.90, TLI = 0.88, RMSEA = 0.081, SRMR = 0.05, which shows construct validity of the tool.

### Positive and negative affect

According to Watson and his colleagues, positive affect involves experiencing emotions such as joy, happiness, excitement, and contentment^41^. Negative affect, on the other hand, encompasses emotions like sadness, anxiety, anger, and fear. It represents the degree to which an individual experiences negative emotions and perceives their life in a less favorable manner^41^ The short version of the positive and negative affect schedule (PANAS-SF) was used to measure positive and negative affect^41^. It was comprised of two components, with 10 items in each. Before being used in the present investigation, the scale was translated into Mandarin and verified. Participants replied on a 5-point Likert scale ranging from 1 (almost none) to 5 (very much). The greater the value, the greater the positive/negative emotion. Total score ranges between 10 and 50 for each dimension. Cronbach alpha value for the scale is 0.87. Further, the tool with one factor model is valid in terms of all significant fit indices such as X^2^/df = 3.10, CFI = 0.97, TLI = 0.96, RMSEA = 0.05, SRMR = 0.041 indicating construct validity of the scale.

### Procedure of the study

The study intends to analyse the mediating effect of life satisfaction, and positive/negative affect for the association among self-efficacy, career exploration and career adaptability. Before starting the research work, ethical approval from the institutional review board of Shaanxi normal university was taken. After all essential approvals, data was collected from adolescents majoring in the field of physical education at Chinese universities. All respondents signed informed consent before participating in the study. Further, the protocol and ethical guidelines outlined in the Declaration of Helsinki and Article 14, Chapter III of the Statistics Law of the People’s Republic of China were rigorously followed throughout the study. The participants were assured that their data would be kept confidential. Those who refused to participate were respected for their decision and were not forced to participate. The convenient sampling technique was used to collect data. A set of questionnaires on the study variables was distributed among all partcipants who consent to participate. Those who refused to sign the informed consent were not inclued in the study. Privacy and confidentiality of the particpants was ensured before data collection. After data collection, the incomplete questionnaires were excluded, and data was checked for missing values. Responses with missing values were removed. Then data analyses was applied by calculating descriptive statistics, bivariate correlation, and developing multiple mediation models in Hayes Process vers. 3.4.1. After analyses, results were generated, discussion was made, and conclusions were drawn.

### Analysis techniques

Data was analyzed by using SPSS ver. 26, and Hayes PROCESS ver. 3.4.1. Descriptive statistics was taken by computing mean values and standard deviations of all variables. Further, bivariate correlation was calculated to see association among study variables. Moreover, confirmatory factor analysis was used to see measurement model and factor structure of the variables. In addition, hypotheses were tested by using Hayes PROCESS to develop multiple mediation models for the mediating effect of life satisfaction, and positive/negative affect between self-efficacy and career adaptability and career exploration. AMOS ver. 24 graphic was used to develop measurement model and validity of instruments.

## Results

### Descriptive statistics and correlation

Table 1 shows descriptive statistics in terms of means and standard deviation. Further, bivariate correlation was computed to see the relationship among study variables such as self-efficacy, life satisfaction, positive/negative affect, career exploration and career adaptability. It is obvious from Table 1 that generalized self-efficacy (r = 0.508, *p* < 0.01), life-satisfaction (r = 0.198, *p* < 0.01), and positive affect (r = 0.199, *p* < 0.01), are positively associated with career exploration. Similarly, career adaptability is also positively associated with self-efficacy (r = 0.322, *p* < 0.01), life-satisfaction (r = 0.420, *p* < 0.01), and positive affect (r = 0.519, *p* < 0.01). On the other hand, negative affect is inversely related with career exploration. (r = − 0.186, *p* < 0.01), and has no association with career adaptability (r = 0.035, *p* > 0.01). Further, gender played significant role in affecting career exploration t(1379) = 2.311, *p* < 0.05), and career adaptability t(1379) = 3.187, *p* < 0.05) of adolescents majoring in physical education. A significant difference between males (M = 28.92, SD = 5.49) and females (M = 28.19, SD = 5.60) in exploring a career emerge. Also, boys (M = 36.1, SD = 9.20) and girls (M = 34.40, SD = 9.93) are significantly different in adapting a career. Hence, gender was controlled for the major analysis.

**Table 1:** Descriptive statistics and correlations among variables.

Variables n = 1381	Mean	SD	1	2	3	4	5	6
1. GSE	32.69	6.72	1	0.224**	0.276**	− 0.151**	0.508**	0.322**
2. LS	20.82	3.69		1	0.463**	− 0.185**	0.198**	0.420**
3. PA	30.94	5.15			1	0.093**	0.199**	0.519**
4. NA	27.20	5.69				1	− 0.186**	− 0.035
5. CE	28.66	5.53					1	0.244**
6. CA	35.51	9.48						1

**Correlation is significant at the 0.01 level (2-tailed). GSE, generalized self-efficacy; LS, life satisfaction; PA, positive affect; NA, negative affect; CE, career exploration; CA, career adaptability.

### Mediation analysis

Mediation model was developed Hayes PROCESS ver. 3.4.1 to see the mediating effect of life satisfaction and positive/negative affect for the association of generalized self-efficacy with career exploration and career adapatbility. 95% biased corrected confidence interval (CI) with 5000 bootstraps was applied for assessing mediation effects. The unstandardized coefficients are shown in the analysis.

**Figure 1:**
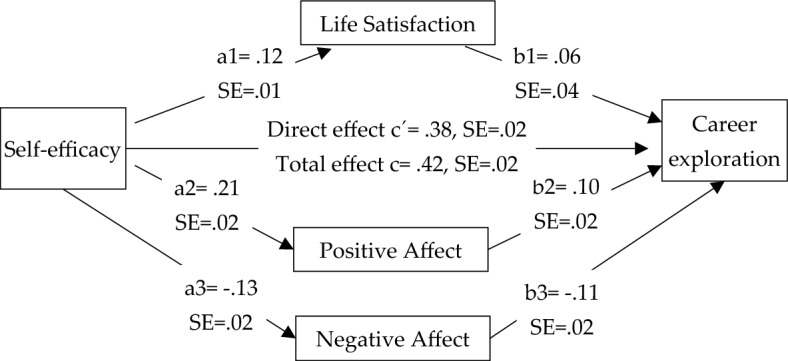
Mediation model for self-efficacy and career exploration.

First, a parallel mediation model for subjective well-being explanatory model of adolescents’ self-efficacy and career exploration was developed. That is, mediation of life satisfaction and positive/negative affect between self-efficacy and career exploration was measured. Multiple mediation model in Fig. 1 shows that self-efficacy positively influences life satisfaction (a1 = 0.12, *p* < 0.001), and positive affect (a2 = 0.21, *p* < 0.001), while inversely influences negative affect (a3 = − 0.13, *p* < 0.001). It means that life satisfaction, and positive affect is increased with enhanced self-efficacy, while negative affect is decreased with an increased self-efficacy. On the other hand, life satisfaction (b1 = 0.06, *p* = 0.093) has no effect on career exploration, while positive affect (b2 = 0.10, *p* = 0.025) has a positive influence on career exploration exhibiting more career exploration with higher positive affect. Further, negative affect is inversely associated with career exploration (b3 = − 0.11, *p* < 0.001) indicating less career exploration with higher negative affect. In addition, self-efficacy has a significant direct effect on career exploration (c′ = 0.382, *p* < 0.001) meaning career exploration is higher when self-efficacy is enhanced (H1). All variables explained 27.75% variance in career exploration.

Moreover, bootstrap analyses indicated that life satisfaction did not play an intermediary effect between elf-efficacy and career exploration (ß = 0.0083, SE = 0.0053, 95% CI = − 0.0014, 0.0195). This does not support the hypothesis that life satisfaction would mediate between self-efficacy and career exploration (H2). On the contrary, the relationship between self-efficacy and career exploration is significantly mediated by positive affect (ß = 0.0138, SE = 0.0062, 95% CI = 0.0021, 0.0264), and negative affect (ß = 0.0141, SE = 0.0039, 95% CI = 0.0073, 0.0225) as there is no zero in the range of confidence interval. Hence, hypothesis that self-efficacy transfers its effect to career exploration through positive/negative affect is supported (H3).

**Figure 2:**
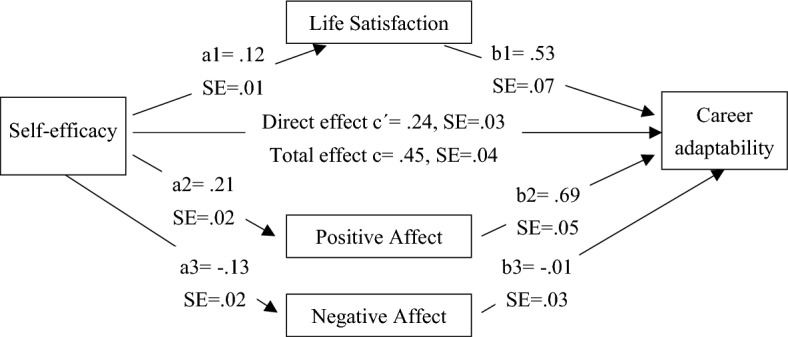
Mediation model for self-efficacy and career adaptability.

Another mediation model for the mediating effect of subjective well-being between self-efficacy and career adaptability was developed. That is, mediation of life satisfaction and positive/negative affect between self-efficacy and career adaptability was measured. Model in Fig. 2 indicates that self-efficacy positively influences life satisfaction (a1 = 0.12, *p* < 0.001), and positive affect (a2 = 0.21, *p* < 0.001), while inversely influences negative affect (a3 = − 0.13, *p* < 0.001). Further, life satisfaction (b1 = 0.53, *p* < 0.001) and positive affect (b2 = 0.69, *p* < 0.001) have a positive influence on career adaptability showing higher life satisfaction and more positive affect leading to better career adaptability. On the other hand, negative affect has no association with career adaptability (b3 = − 0.01, *p* = 0.7852). In addition, self-efficacy has a significant direct effect on career adaptability (c′ = 0.24, *p* < 0.001) showing better career adaptability with higher self-efficacy (H4). All variables explained 33.75% variance in career adaptability.

Moreover, bootstrap analyses indicated that the relationship between self-efficacy and career adaptability is significantly mediated by life satisfaction (ß = 0.0652, SE = 0.0154, 95% CI = 0.0370, 0.0972) supporting H5. Further, positive affect significantly mediated between self-efficacy and career adaptability (ß = 0.1464, SE = 0.0213, 95% CI = 0.1069, 0.1912) as there is no zero in the range of confidence interval. On the other hand, negative affect did not play an intermediary effect between elf-efficacy and career adaptability (ß = 0.0013, SE = 0.0060, 95% CI = − 0.0110, 0.0130) as CI range has a zero. Hence, hypothesis that self-efficacy transfers its effect to career adaptability through positive/negative affect is partially supported as positive affect played a significant intermediary role between the two, but negative affect did not. The results partially support H6.

### Discussion

Contributing career construction theory, the present study authenticates a theoretical model indicating the relationship of generalized self-efficacy with physical education students’ career development through the mediation of components of subjective well-being such as life-satisfaction, and positive/negative affects. Our study’s model indicates the significance of a theoretical framework for forecasting physical education students’ career exploration and career adaptability in China. Furthermore, utilizing generalized self-efficacy, life-satisfaction, positive/negative affects, career exploration, and career adaptability in a multiple mediation model are new aspects in comparison to the previous investigations.

### Effect of self-efficacy, life-satisfaction, and positive/negative affect on career exploration

First, it was hypothesized that self-efficacy would positively affect career exploration. The results indicated that self-efficacy was directly connected with physical education students’ career exploration. Regarding the correlation between self-efficacy and career exploration, both theoretical and empirical research has shown consistent results. For instance, SCCT suggests that ndividuals with high self-efficacy are more inclined to establish ambitious career objectives and persevere when faced with obstacles (Lent et al. 1994). Lent et al. (2000) conducted a study that revealed a positive correlation between self-efficacy and both career exploration and career decision-making self-efficacy in college students, emphasizing the significance of self-efficacy in career development. A number of empirical studies^10,15,16,42^ and the social cognitive career theory^12–14^ confirmed that self-efficacy has a significant positive impact on career exploration of adolescents. The results are in line with empirical research and a meta-analysis which has established positive association between self-efficacy and career exploration of adolescents^18,42^. Hence, self-efficacy increases the chances of better career choice or exploration leading to better career development. It implies that the organizations, educational institutions, and career counseling services can design and implement tailored career development programs that specifically focus on enhancing individuals’ self-efficacy beliefs.

Further, it isnoticed from the results that self-efficacy positively predicts life satisfaction. Supporting the above statement theoretically, SCCT suggests a link between self-efficacy and subjective well-being, indicating that individuals who have confidence in their abilities to achieve their goals often experience higher levels of satisfaction and happiness in life (Lent and Brown 2013). Lent and Brown (2006) conducted research that showed a positive correlation between self-efficacy and both job satisfaction and life satisfaction, providing evidence for the role of self-efficacy in enhancing subjective well-being. According to the perspective of several other authors, self-efficacy determines better perceptions towards life^43–45^. It means that if students have higher self-efficacy, they would have a happier and more fulfilling life. Another important finding of this study is that self-efficacy instils in students the positive attitude, and reduces the negative perspectives among them. It confirms that possessing self-efficacy is beneficial for adopting positivity and avoiding negative affect^20^. It implies that educators can empower students to succeed in their career, develop resilience, and navigate life’s challenges with confidence and positivity. Further, interventions aimed at enhancing self-efficacy may not only lead to improved career outcomes but also contribute to overall life satisfaction and happiness. This suggests that programs and interventions designed to boost self-efficacy could have broader positive impacts on individuals’ well-being beyond just their professional lives.

Moreover, we have pointed out that high levels of life satisfaction and positive affect lead to better career exploration, while negative affect inhibits the ability to explore career among Chinese adolescents. According to SCCTi, ndividuals who pursue careers that provide opportunities for growth and development are more likely to experience a sense of fulfillment and purpose, and those who find fulfillment and purpose in their work are often motivated to seek out opportunities for growth and development in their careers suggesting a mutually reinforcing cycle where career growth and development contribute to a sense of fulfillment and purpose, which in turn motivates further career development (Lent and Brown 2013). Related empirical research also shows that there is a positive association between life satisfaction, positive affect and career exploration^20,42,46^, whereas there is a negative association between negative affect and career exploration^47,48^. Higher life satisfaction and positive attitude is associated with higher career exploration, supporting the finding from empirical research that adolescents with high life satisfaction and healthier emotional condition would be better in career exploration^20^. In contrast, having negative emotions or expressions lead to poor career exploration as negative views or emotions are associated with lower career development^47,48^. The results imply that educational institutions, counseling services, and youth development programs can incorporate emotional intelligence training into their curriculum. The type of interventions may help them cultivate higher levels of life satisfaction, positive affect, and emotional resilience which, in turn, can facilitate better career exploration by enabling adolescents to approach decision-making processes with a positive mindset, manage stress and setbacks more effectively, and engage in proactive exploration of career options.

### Mediation of life-satisfaction, and positive/negative affect between self-efficacy and career exploration

One key conclusion of our work is the hypothesized relationship between self-efficacy and career exploration through life satisfaction and positive/negative affect. In the Chinese setting, we expected to explore mediation of life satisfaction and positive/negative affect between self-efficacy and career exploration. The relationship gives a novel viewpoint about the mediation model. Positive/negative affect played a significant mediating role complementing previous finding that self-efficacy decreases negative emotions^20^ and that negative affect leads to decreased career exploration^47,48^ providing a base for mediation of positive/negative affect. However, the unusual aspect is life satisfaction does not play a mediating role between the self-efficacy and career exploration. This contradicts prior research in which life satisfaction had a role as mediators between self-efficacy and career exploration and decreases negative emotions and expressions^20^, thus, leading to better career exploration^18,20,42^. An explanation can be that Chinese being in transition from collectivistic to individualistic behavior, may give greater attention to self-system such as self-efficacy in their pursuit of happiness^49,50^. Another explanation is that individuals who perceive self-efficacy to be more important for their well-being, and are aware of the importance of having good self-perceptions, and positive emotions or energy, have better career exploration. It can also be explained as self-efficacy may have such a strong direct effect on career exploration that the influence of life satisfaction becomes relatively negligible. Individuals with high self-efficacy may be more proactive and confident in exploring career options regardless of their overall life satisfaction.

### Effect of self-efficacy, life-satisfaction, and positive/negative affect on career adaptability

The results suggested that self-efficacy was both directly and indirectly related to career adaptability among Chinese physical education students. The results of a previous study are associated with the current finding that there is positive relationship between self-efficacy and career adaptability^51,52^. Social cognitive career theory^12–14^, and a meta-analyses^17^ supported that self-efficacy has a positive effect on career adaptability. Hence, self-efficacy raises the likelihood of improved career adaptability, resulting in enhanced career growth.

Furthermore, life satisfaction is positively associated with career adaptability. Previous literature has provided evidence on the positive association between life satisfaction and quality career adaptability^52–55^. It implies that if students had higher life satisfaction, their lives would be better and more satisfying, and they could achieve greater career adaptability.

In addition, we have found that individuals with positive emotions and energetic attitude would better adapt to their career, but those with negative emotions would have poor career adaptability as negativity hinders the potential. According to earlier research, there is a positive relationship between life satisfaction, positive affect, and career adaptability^52,56^, but a negative relationship between negative affect and career adaptability^57^. Marcionetti and Rossier^52^ reported that the adolescents with higher life satisfaction would be better at career adaptability indicating greater life happiness and positive attitude connected with increased career adaptability. Negative emotions or expressions are connected with less career growth^57,58^ while negative emotions or expressions are associated with less career development.

### Mediation of life-satisfaction, and positive/negative affect between self-efficacy and career adaptability

Another important conclusion of our work is the hypothesized indirect relationship between self-efficacy and career adaptability. This indirect relationship gives a novel perspective. In the Chinese setting, we expected to explore mediation of life satisfaction and positive/negative affect between self-efficacy and career adaptability. We have found that self-efficacy is connected with life satisfaction, positive which, in turn, is associated with career adaptability of Chinese students who belong to sports field. Further, self-efficacy and career eadaptability were significantly mediated by life satisfaction and positive affect. This indicates that individuals with higher levels of self-efficacy are more likely to experience greater life satisfaction and positive emotions, which in turn facilitate their ability to adapt and thrive in their careers. This complements prior research in which life satisfaction and positive affect had a role as mediators between self-efficacy and career development^20^. Another research also authenticates the current fidning that self-efficacy improves life satisfaction and positive affect^20,43–45^, thus, leading to better career adaptability^17^. On the other hand, negative affec did not mediate between self-efficacy and career adaptability suggesting that feelings of negativity may not impede individuals’ capacity to adapt in their careers as strongly as positive emotions enhance it. Previous research indicates contradicting results that self-efficacy decreases negative emotions^20^ and that negative affect leads to decreased career adaptability^57,58^. The above results results accentuate the importance of considering the role of both cognitive and affective factors in shaping individuals’ career adaptability. The implications could be that the organizations and career development programs may benefit from incorporating interventions that target both self-efficacy enhancement, and the promotion of life satisfaction and positive emotional experiences, as these factors are crucial for fostering greater career adaptability among individuals. Additionally, efforts to mitigate negative affect may not be as critical for enhancing career adaptability, although addressing negative emotions can still be valuable for overall well-being and job satisfaction.

In addition to all findings, it is noticed that a significant difference between males and females for both career exploration and career adaptability. Mean values on career exploration and career adaptability were higher for boys, meaning that male students were better at career development than female students. That is consistent with prior research that revealed a significant gender difference for career exploration^19^, and career adaptability^32,33^. It suggestes that future interventions and support system may consider gender disparities for enhancing career exploration and career adaptability among individuals. Educational institutions, career counseling services, and employers can implement initiatives aimed at empowering female students and professionals to explore diverse career paths and enhance their adaptability. This could include mentorship programs, workshops on confidence-building and decision-making skills, and efforts to challenge gender stereotypes and biases in career guidance and hiring practices. By addressing these disparities and promoting equal opportunities for career development, organizations can foster a more inclusive and diverse workforce, ultimately benefiting both individuals and society as a whole.

### Research implications

The study’s findings imply that self-efficacy leads to a better career development by increasing life satisfaction, improving positive emotions, and reducing the negative consequences. These findings are in agreement with the social cognitive career theory^12–14,34^ in relating subjective well-being to self-efficacy and career related behaviors or career development. Research findings demonstrate that self-efficacy is directly and indirectly associated with career development providing evidence of the role of components of subjective well-being as mediators in the association of self-efficacy with sports adolescents’ career development.

There are several implications of the study which drew our attention to the significance of self-efficacy in enhancing subjective well-being and career development. The findings imply that physical education students should be encouraged to enhance their self-efficacy for career development and devising future interventions. Institutions should examine the professional development of physical education teachers in the area of promoting self-efficacy, increasing life satisfaction, promoting positive behavior and preventing negative attitude among physical education students. Future study in different cultures that focuses on the mediation of other aspects of well-being between self-efficacy and career development may aid in the comprehension of novel mechanisms influencing this causal association. Consideration might be given to conduct further intervention studies aimed at developing career related behaviors using self-efficacy.

### Research limitations

This study has strengths and benefits for theory and practice, but future research have to address some shortcomings. In this study, the model was evaluated on the basis of quantitative data, which might be challenged for sampling bias. Thus, we recommend a qualitative technique and an in-depth interview for future research to triangulate the findings. A further restriction is that the research was done in Xi’an province and Xinjiang Uygur Autonomous Region, China, using a convenient sampling method. This may result in sample bias, and the results may be generalizable in these particular provinces only. Future study may collect data from physical education students in other parts of the country and internationally to provide more generalizable findings.

Another limitation is that self-report assessments tools were used, which may create inflated correlations. In the future, experimental designs may be used for study. In addition, the sample for this study includes a homogenous group of physical education students and taking a diverse sample may produce distinct results. Importantly, we have evaluated components of subjective well-being as discrete, independent, mediating factors. For a better knowledge of the mediators’ effect, they might be taken with a wider range. For instance, individuals with lower life satisfaction and positive attitude and higher negative behavior, high life satisfaction, high positive attitude and low negative emotions, high life satisfaction, high positive attitude and high negative emotions, low life satisfaction, low positive attitude and low negative emotions shold be examined. Similarly, self-efficacy and career development have been evaluated as distinct independent variables. Samples from a population with a wider range of levels of self-efficacy and different attitude of career development may aid in understanding the relationship among these factors in this particular area.

## Conclusion

This research describes research on the mediating effect of components of subjective well-being (life satisfaction, positive affect, and negative affect) between self-efficacy and career development in physical education students under the theoretical framework of the career construction theory. The research model for this study rely on data collected from 1381 physical education students at Chinese universities. We developed a multiple mediation model by using Hayes process and determined that self-efficacy directly and indirectly predicted career development among physical education students. Self-efficacy increases life satisfaction and positive affect and decreases negative emotions. Positive affect and negative affect played significant mediating role between self-efficacy and career exploration while life-satisfaction did not. Conversely, life satisfaction and positive affect significantly mediated between self-efficacy and career adaptability and negative affect did not. The research indicated that students who have higher self-efficacy will have more positive affect and less negative behavior leading to better career exploration. Conversely, those who have higher self-efficacy would have more life satisfaction and positive affect leading to better career adaptability. The study implies that students’ self-efficacy should be enhanced, positive behavior should be instilled, negative behavior should be reduced and life satisfaction should be increased to construct better career development.

## Data Availability

The datasets used and/or analysed during the current study are available from the corresponding author upon reasonable request.
